# Changes in root‐exudate‐induced respiration reveal a novel mechanism through which drought affects ecosystem carbon cycling

**DOI:** 10.1111/nph.16001

**Published:** 2019-07-24

**Authors:** Franciska T. de Vries, Alex Williams, Fiona Stringer, Robert Willcocks, Rosie McEwing, Holly Langridge, Angela L. Straathof

**Affiliations:** ^1^ School of Earth and Environmental Sciences The University of Manchester Oxford Road Manchester M13 9PT UK; ^2^ Institute for Biodiversity and Ecosystem Dynamics University of Amsterdam PO Box 94240 Amsterdam 1090 GE the Netherlands

**Keywords:** carbon, climate change, drought, plant–soil interactions, root exudate, root traits, soil bacteria, soil fungi

## Abstract

- Root exudates play an important role in ecosystem response to climate change, but the functional consequences of drought‐induced changes in the quality of root exudates are unknown. Here, we addressed this knowledge gap in a unique experimental approach.
- We subjected two common grassland species that differ widely in their growth strategies and root systems, the grass *Holcus lanatus* and the forb *Rumex acetosa*, to 2 wk of drought. We collected root exudates and soils at the end of the drought and after 2 wk of recovery and readded all root exudates to all soils in a fully reciprocal set‐up to measure root‐exudate‐induced respiration.
- We found that soil treatment was unimportant for determining root‐exudate‐induced respiration. By contrast, root exudates collected from plants that had experienced drought clearly triggered more soil respiration than exudates from undroughted plants. Importantly, this increased respiration compensated for the lower rates of root exudation in droughted plants.
- Our findings reveal a novel mechanism through which drought can continue to affect ecosystem carbon cycling, and a potential plant strategy to facilitate regrowth through stimulating microbial activity. These findings have important implications for understanding plant and ecosystem response to drought.

## Introduction

Ecologists increasingly recognize the role of plant root exudates in ecosystem functioning. Root exudates – complex, soluble mixtures of carbon (C) compounds, such as sugars, amino acids, organic acids, and secondary metabolites – can comprise up to 10% of plant photosynthate and are a highly dynamic source of C input into the soil (Jones *et al*., 2004, 2009). Despite early acknowledgement of the role of root exudates in, for example, phosphorus (P) solubilization (Hoffland *et al*., 1989) and the priming of soil organic matter decomposition (Kuzyakov *et al*., 1999), root exudation has long been considered to be a passive process (Jones *et al*., 2009). However, from an evolutionary perspective, it is hard to explain why plants would leak substantial amounts of a key resource into the soil. Plant C and nutrient exchange with mycorrhizal fungi has long been explored from a cost–benefit perspective, and plants have been shown to regulate their C allocation to mycorrhizal fungi in response to changes in environmental conditions and nutrient supply by the fungus (Fellbaum *et al*., 2012; Arguello *et al*., 2016; Ji & Bever, 2016). Similarly, it has been hypothesized that plants and heterotrophic soil microbes have coevolved and that root exudates are one of the main pathways of providing C for microbial growth and, hence, play a vital role in plant–microbe communication (Mendes *et al*., 2013; Mommer *et al*., 2016).

Accumulating evidence indicates root exudation is an active process that can positively feed back to plant performance. For example, grazing can promote root exudation in grassland species, which stimulates the soil microbial community, subsequently improving plant regrowth through increasing nitrogen (N) mineralization (Hamilton & Frank, 2001). Similarly, elevated atmospheric CO_2_ concentrations can increase root exudation rates, which can in turn trigger a feedback of increased microbial N mineralization rates, potentially alleviating N limitation under these conditions (Phillips *et al*., 2011). Although these studies focused on changes in the quantity of root exudation, later studies tend to focus on changes in the quality (i.e. chemical composition) of root exudation. Recent work shows that plants can actively alter the composition of their root exudates, and that these changes in the relative abundance of specific compounds have implications for the composition of rhizosphere soil microbial communities (Zhalnina *et al*., 2018). Moreover, root‐exudate‐induced changes in rhizosphere microbial communities can feed back to alter plant phenology: changes in rhizosphere microbes increased the availability of N and downregulated genes that trigger flowering, thus stimulating further plant growth and potentially increasing reproductive fitness (Lu *et al*., 2018).

It is well known that drought can strongly affect plant growth and soil microbial community activity and composition, resulting in altered rates of C and N cycling processes (Gordon *et al*., 2008; Sanaullah *et al*., 2011; De Vries *et al*., 2016, 2018; de Nijs *et al*., 2019). Similar to elevated CO_2_ and grazing, root exudates have been hypothesized to play an important role in the response of soil microbial communities to drought, but it is challenging to disentangle root‐exudate‐mediated effects from other mechanisms through which drought affects soil microbial communities. Drought directly affects soil fungal and bacterial communities, but the rate of root exudation is also affected by drought; both mechanisms can result in altered heterotrophic respiration rates. For example, ^13^CO_2_ pulse labelling studies have found that drought reduces the respiration and transfer to soil microorganisms of recently fixed C (Ruehr *et al*., 2009; Fuchslueger *et al*., 2014). However, it is impossible to assess whether altered quantity or quality of root exudation, changes in microbial C use efficiency, or altered rates of (autotrophic) root respiration are driving these patterns. Despite reductions in plant growth and a general decrease of C transfer belowground, a number of studies have also found increases in rhizodeposition (which includes root exudation, but also mucilage, sloughed‐off border cells, and senescence; Jones *et al*., 2009) in response to drought, especially when expressed per unit plant biomass (Preece & Penuelas, 2016). These workers suggested that increased rates of rhizodeposition might help roots move through dry soil by improved lubrication, and/or result from increased root mortality and cell damage under drought (Hasibeder *et al*., 2015).

Drought not only changes the quantity of root exudates, but also their quality. By collecting root exudates from (part of) the intact root system in solution, many studies have shown that drought affects the composition of root exudates. For example, in a pot experiment where root exudates were collected from washed root systems of sunflower and soybean at the end of a 2‐wk drought period, soybean root exudates showed a clear shift in metabolite composition, whereas sunflower increased rates of root exudation (Canarini *et al*., 2016). Similarly, Gargallo‐Garriga *et al*. (2018) found that drought increased the concentration of secondary metabolites in root exudates of holm oak, whereas during recovery after drought the root exudates were dominated by primary metabolites. An earlier experiment found that maize (*Zea mays*) plants that were hydroponically subjected to osmotic stress had an increased concentration of organic acids in root exudates, which was hypothesized to provide an adaptation to drought by increasing the solubilization of soil nutrients (Song *et al*., 2012). However, the functional implications of drought‐induced changes in root exudate quantity and quality have never been assessed.

Here, we hypothesized that drought‐induced changes in root exudation have implications for soil functioning. We assessed patterns of root exudation, and their response to drought, and the implications for soil respiration, across two common temperate grassland species that differ widely in their growth strategies. *Holcus lanatus* L. is a fast‐growing grass with a root system that consists of long, thin roots of low tissue density (i.e. high specific root length (SRL) and low root tissue density (RTD) and root dry matter content (RDMC); De Vries & Bardgett, 2016), and which has been shown to have high C allocation to the rhizosphere (Ladygina & Hedlund, 2010). *Rumex acetosa* L. is a nonmycorrhizal, fast‐growing forb with thicker, denser roots of relatively low SRL, high RTD, and high RDMC (De Vries & Bardgett, 2016). Thus, we hypothesized that *H. lanatus* would have higher rates of root exudation that would trigger more respiration. Moreover, we hypothesized that, across the two species, root exudates from droughted plants would increase soil heterotrophic respiration. This could be a consequence of changes in the concentration of primary and secondary metabolites, which might stimulate whole‐community microbial respiration through a larger proportion of sugars, and stimulate or suppress certain groups of microbes, respectively. Alternatively, an increase in the concentration of organic acids might result in priming of soil organic C decomposition (Falchini *et al*., 2003; Brant *et al*., 2006). We used a unique approach where we collected root exudates from droughted and control plants and readded these to the soils that the droughted and control plants had grown in, which allowed us to disentangle the effect of changes in soil microbial communities and changes in root exudates on root‐exudate‐induced respiration.

## Materials and Methods

### Experimental set‐up

Mesotrophic grassland soil (pH 5.35 ± 0.3, total N 0.57 ± 0.02%, total C 5.70 ± 0.14%) was collected in November 2016 from extensively managed grassland in the Ingleborough Nature Reserve in the Yorkshire Dales, UK (54°11′38.7′′N, 2°20′54.4′′W), sieved to 4 mm mesh size, and kept in the fridge until the start of the experiment. We used two common temperate grasslands species that differ widely in their growth strategies, and consequently in their root traits: the grass *H. lanatus* (hereafter *Holcus*) and the forb *R. acetosa* (hereafter *Rumex*). Seeds for both species were purchased from Emorsgate Seeds (King's Lynn, UK) and germinated in seed trays using the field soil collected. After 2 wk, one individual of each species was planted in each 500 ml pot (height 8 cm, diameter 10.5 cm) filled with 350 g (FW) field soil, and grown for 10 wk until the start of the experiment on 29 January 2017. For each species, a set of pots was then subjected to a 2 wk drought treatment and a well‐watered treatment. For drought pots, watering was ceased until a soil moisture content of 20% of the water‐holding capacity (WHC) was reached, after which the pots were kept at that weight until the end of the 2 wk drought period, whereas control pots were watered every 2–3 d to a moisture content of 60% WHC (Supporting Information Fig. S1). At the end of the drought, one set of drought and control pots was harvested (end‐of‐drought harvest). The second set were carefully rewetted to 60% WHC and allowed to recover for 2 wk before a final harvest (2‐wk recovery harvest). With five replicates per treatment, this resulted in 40 pots (two species × two treatments × two harvests × five replicates); pots were arranged in a randomized block design in a controlled growth chamber (Percival AR‐66L2) with a daily cycle of 16 h of light at 21°C followed by 8 h of darkness at 16°C.

**Figure 1:**
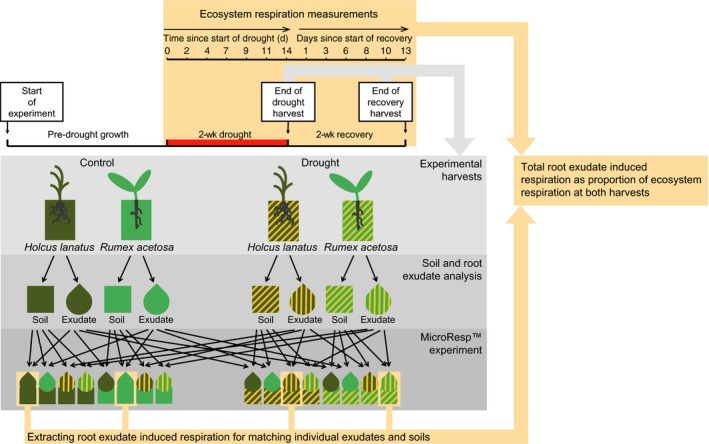
Schematic representation of the experimental set‐up of the main experiment and the MicroResp^™^ experiment. For each experimental harvest (end of drought and 2‐wk recovery) all individual soils and root exudates from *Holcus lanatus* and *Rumex acetosa* were collected, analysed, and used for the subsequent MicroResp experiment.

Ecosystem respiration was measured every 3 d from 1 d before the start of the drought to the final harvest 2 wk after the drought had ended using an infrared gas analyser (EGM‐5; PP Systems, Amesbury, MA, USA) and a blacked out, air‐tight chamber. At each of the two harvests, plants were carefully removed from the pots and 50 g of root‐adhering soil was collected, sieved to 2 mm, and used for soil nutrient analyses (under controlled laboratory conditions (18°C)) and assessment of root‐exudate‐induced respiration (see Fig. 1 for a schematic representation of the experimental set‐up). The roots of intact plants were then used for root exudate collection and subsequent analysis or root‐exudate‐induced respiration, after which aboveground and belowground plant parts were separated and analysed for biomass, C and N content, and morphological and structural root traits.

### Root exudate collection

Roots of intact plants were carefully washed to remove all remaining soil, and dead roots were removed using stainless steel tweezers. Each individual plant was then transferred to a hydroponics solution, which consisted of 100 ml soil solution obtained from a 1 : 10, soil : Milli‐Q water mixture, and rearranged in the controlled growth chamber for 48 h. The purpose of this 48 h period in hydroponic solution was to avoid collecting leaked root cell contents as a result of damage done by root washing as well as through rewetting of dry plant roots (as reviewed in Oburger & Jones, 2018). After 48 h, each plant was rinsed of hydroponics solution, transferred to a flask with 100 ml sterile Milli‐Q water (placed on ice) and shaken at 60 rpm at 18°C and ambient light for 2 h to collect root exudates. An 80 ml sample of each root exudate solution was filter‐sterilized to 0.22 μm, freeze‐dried, and stored at −80°C until use for the root‐exudate‐induced respiration experiment.

### Plant analyses

After exudate collection, plant roots and shoots were separated, and shoots were dried at 60°C for 48 h, weighed, and ground for C and N analysis on a Vario EL elemental analyser (Elementar, Hanau, Germany). FW measurements of roots were taken before storage in 10% ethanol until root morphological analyses were conducted. Root systems were then scanned on an Epson flatbed scanner and analysed for morphological root traits using the winrhizo ^®^ root analysis software (Regent Instruments Inc., Quebec, QC, Canada). After analysis, roots were dried at 60°C for 48 h, weighed, and ground for analysis of root C content and N content (RNC) on an Elementar Vario EL elemental analyser. SRL was calculated by dividing the total root length by the dry biomass (cm g^−1^). RTD was calculated by dividing the weight of the dry biomass by the root volume (g cm^−3^). RDMC was calculated as DW divided by FW.

### Soil analyses

Plant‐available nitrate and ammonium were extracted by shaking 5 g of soil in 25 ml 1 M potassium chloride for 1 h, filtered (Whatman 42), and analysed on an AA3 Auto Analyser (Seal Analytical, Southampton, UK). Total dissolved N and dissolved organic C (DOC) were extracted by shaking 5 g of soil in 30 ml Milli‐Q for 10 min, filtered (0.45 μm), and analysed for total N using an Auto Analyser (Seal Analytical) and for total organic C (TOC) using a Shimadzu 5000 A TOC analyser (Japan). Microbial biomass C and N were measured using the chloroform fumigation extraction method as described by Brookes *et al*. (1985). Briefly, 5 g of fumigated and unfumigated soil samples were extracted in 25 ml of 0.5 M potassium sulphate by shaking for 30 min and filtering through Whatman 42 filter paper. DOC and total N were measured by a Shimadzu 5000 A TOC analyser and an Auto Analyser (Seal Analytical), and microbial C and N were calculated as the difference between fumigated and unfumigated samples, using a *k*_EN_ of 0.54 (Brookes *et al*., 1985) and a *k*_EC_ of 0.35 (Sparling *et al*., 1990). All measures done on fresh soil were calculated per gram dry soil, as determined gravimetrically after 24 h oven drying at 105°C.

Soil microbial communities were characterized by analysing phospholipid fatty acids (PLFAs), which were extracted from 0.5 g of freeze‐dried soil in Bligh–Dyer extractant, containing an internal 19:0 phosphatidylcholine standard (Avanti Polar Lipids, Alabaster, AL, USA), for 2 h rotating end over end. The liquid phase was then collected after centrifugation, 1.0 ml chloroform : water (1 : 1) was added, and the lower phase siphoned off and used for lipid separation. Lipids were separated by solid phase extraction (SPE) using a 96‐well SPE plate (50 mg silica; Sigma‐Aldrich, St Louis, MO, USA) with chloroform, acetone, and 5 : 5 : 1, methanol : chloroform : water. Fatty acids were then transesterified and extracted and analysed on a GC 7890B (Agilent Technologies, Santa Clara, CA, USA). We used 20 microbial‐specific PLFAs in subsequent data analysis. The fatty acids i15:0, a15:0, i16:0, and i17:0 were used as biomarkers for Gram‐positive bacteria; 16:1ω7, 18:1ω7, cy17:0, and cy19:0 were used as biomarkers of Gram‐negative bacteria; 15:0 and 17:0 were used as general bacterial markers (Frostegård *et al*., 1993); and 18:1ω9 was used as a marker for eukaryotes (Findlay, 2004). Gram‐positive, Gram‐negative, and general bacterial markers were summed to give total bacterial PLFA, and 18:2ω6,9 was used as a marker of fungi (Bååth, 2003; Bååth & Anderson, 2003).

### Root‐exudate‐induced respiration

Assessment of root‐exudate‐induced respiration across all root exudate and soil treatments was conducted using the MicroResp^™^ procedure, modified from Campbell *et al*. (2003). Instead of standard C substrates, we added the root exudates collected from each individual plant from each individual treatment to each individual soil from each individual treatment. This allowed us to assess the relative importance of changes in root exudate quality and quantity, as well as the change in soil properties, in response to the experimental treatment, for root‐exudate‐induced respiration.

Individual freeze‐dried root exudates were resuspended in 6 ml of Milli‐Q water, and 25 μl of this solution from each individual plant (total amount of C in this solution varied from 0.17 to 1.59 μg) was added to *c*. 0.3 g (ranging from 0.14 to 0.42 g) of each individual soil sample in deepwell plates. This was done using eight replicates of each soil randomized across the columns of the deepwell plates, so that each soil replicate was placed within an eight‐well row of the 12 × 8 well plate. Milli‐Q water was added to each soil sample in an additional separate eight‐well row as a negative control. This set‐up resulted in 35 deepwell plates per harvest. MicroResp^™^ assays were conducted over a 5 d period after each harvest, where one experimental block from the root exudate collection was processed per day. The deepwell plates with soil and root exudates were sealed to detection plates using specialized silicone rubber gaskets and then secured with clamps. These were stored in the dark at 21°C for 6 h before detection plates were detached from the deepwell plates and the absorbance read in the spectrophotometer at 570 nm. Plates were then resealed together and stored for a further 16 h to be read again. CO_2_ production rates (μg CO_2_‐C g^−1^ h^−1^) were calculated following Campbell *et al*. (2003) and normalized by the water controls for each soil treatment and species to ensure respiration rates were substrate specific. Resuspended root exudates were analysed for C content by Segmented Flow Analysis TOC at Chemisch Biologisch Laboratorium Bodem in Wageningen, the Netherlands, and used to standardize CO_2_ production rates per microgram root exudate C added. These water‐ and C‐normalized CO_2_ production rates allowed us to assess the effects of changes in root exudate quantity and quality simultaneously. Our 6 and 22 h data roughly showed the same results, but patterns were clearer in the 6 h data; thus, we used the 6 h data for subsequent calculations and analyses.

We estimated the contribution of root‐exudate‐induced respiration to total ecosystem respiration. We used root‐exudate‐induced respiration (µg C per µg^–1^ root exudate C g^–1^ soil h^–1^) from samples with matched root exudate origin and soil origin, together with the absolute amount of root exudate released by the individual root system from that experimental unit (µg h^–1^), to calculate total root‐exudate‐induced respiration. We then expressed this as a percentage of ecosystem respiration measured in that same experimental unit on the date closest to root exudate collection.

### Statistical analyses

Before fitting any statistical model, we examined all data for normality and log‐transformed where necessary. To analyse the data from the main experimental harvests, we fitted three‐way ANOVA models with fixed effects for plant species, drought treatment, and harvest and a random effect for block (aov function in R). Ecosystem respiration measurements from the main experiment were analysed with a repeated‐measures ANOVA model with fixed factors for plant species, drought treatment, and date and a random factor term with experimental unit nested in block and block nested in date.

We analysed root‐exudate‐induced respiration for each harvest separately by linear mixed‐effects models, using the lmer function in the package lme4 to facilitate for noncrossed random effects terms. Specifically, our models included a random term consisting of three noncrossed random effects: soil–root exudate combination nested in experimental unit that soil originated from nested in block, experimental unit that exudate originated from nested in block, and deepwell plate row nested in deepwell plate. We first tested the effect of root exudate C added on water‐normalized respiration rates. Then, we fitted the full model for C‐standardized respiration rates with fixed‐effects soil species, soil treatment, exudate species, and exudate treatment to disentangle the role of soil conditioning by the two species, soil drought history, root exudate species, and root exudate drought history, on root‐exudate‐induced respiration. All analyses were done with R v.3.3.2 (R Core Team, 2016) – code and full model output are available in Tables S1 and S2.

## Results

### Drought and species‐specific effects on plant and soil microbial properties

**Figure 2:**
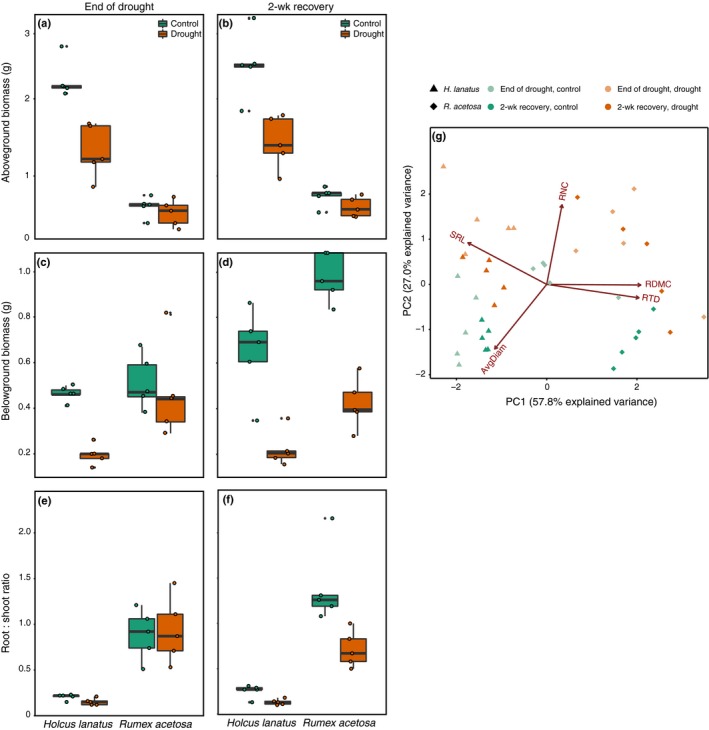
(a, b) Aboveground biomass, (c, d) belowground biomass, (e, f) root : shoot ratio, and (g) root traits for end of drought and post‐recovery harvests as affected by species identity and drought. Lines in boxes represent median, top and bottom of boxes represent first and third quartiles, respectively, and whiskers represent 1.5 interquartile range; data points on principal component analysis plot represent single observations. SRL, specific root length; RNC, root nitrogen content; RDMC, root dry matter content; RTD, root tissue density; AvgDiam, average diameter. See the Results section for statistics.

In both *Holcus* and *Rumex*, drought strongly reduced aboveground and belowground biomass. Drought reduced the aboveground biomass of *Holcus* more than that of *Rumex* (species × treatment interaction *F*_1,28_ = 27.1, *P *<* *0.0001; Fig. 2), and the reduction in root biomass of *Rumex* was stronger at the 2 wk recovery harvest than at the end‐of‐drought harvest (species × treatment × harvest interaction *F*_1,28_ = 4.5, *P *=* *0.041; Fig. 2). *Rumex* had a higher ratio of root‐to‐shoot biomass, and this property was reduced by drought across the two species and harvests, except for *Rumex* at the end‐of‐drought harvest (species × treatment × harvest interaction *F*_1,28_ = 5.5, *P *=* *0.027; Fig. 2). The two species also differed strongly in their root traits, with *Rumex* having higher RTD and RDMC, and *Holcus* having higher SLA. In both species and at both harvests, drought increased RNC (ANOVA main effect of treatment *F*_1,28_ = 41.7, *P *<* *0.0001; Fig. 2g). Effects of drought on other root traits were weaker: drought increased SRL and RDMC, and decreased root diameter (main effect of treatment *F*_1,28_ = 6.48, *P *=* *0.016, *F*_1,28_ = 8.43, *P *=* *0.007, and *F*_1,28_ = 35.8, *P *<* *0.0001, respectively; Fig. 2).

**Figure 3:**
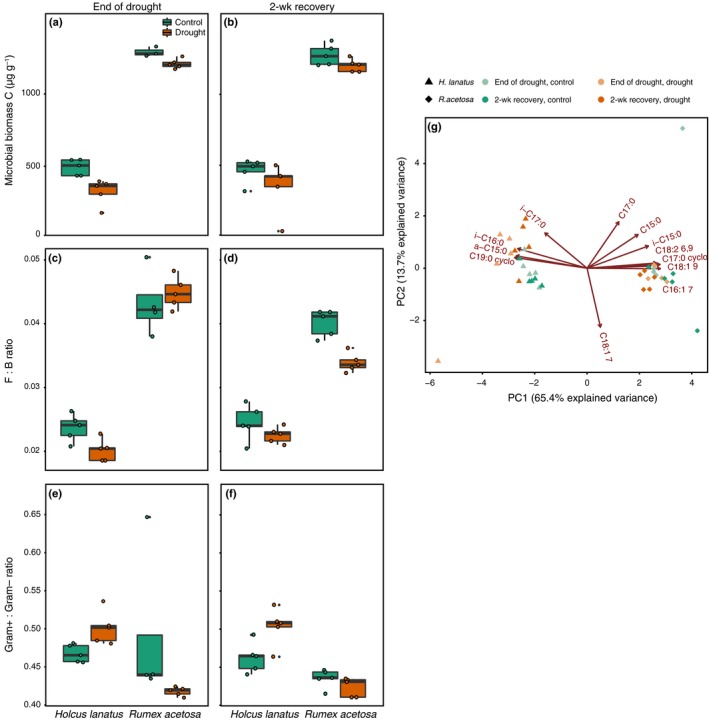
(a, b) Microbial biomass, (c, d) fungal‐to‐bacterial (F : B) phospholipid fatty acids (PLFAs) ratio, (e, f) Gram‐positive bacteria : Gram‐negative bacteria ratio, and (g) microbial PLFAs for end of drought and 2‐wk recovery harvests as affected by species identity (*Holcus lanatus* and *Rumex acetosa*) and drought. Lines in boxes represent median, top and bottom of boxes represent first and third quartiles, and whiskers represent 1.5 interquartile range; data points on principal component analysis plot represent single observations. For individual PLFAs, see the Materials and Methods section. See the Results section for statistics.

Similarly, the two species selected for distinct microbial communities: communities under *Rumex* had higher relative abundances of fungi and eukaryotes than under *Holcus* (Fig. 3g). Microbial biomass C, total PLFA, fungal PLFA, and bacterial PLFA were all at least twice as high under *Rumex* than under *Holcus* (Table 1). By contrast, Gram‐positive and Gram‐negative PLFAs were highest under *Holcus* (Table 1). All these properties were reduced under drought independent of species identity or harvest, but the ratio of fungal‐to‐bacterial PLFA was reduced more by drought under *Rumex* at the 2 wk recovery harvest (species × treatment × harvest interaction *F*_1,27_ = 9.7, *P *=* *0.004; Fig. 3). Whereas drought reduced both Gram‐positive and Gram‐negative PLFAs (Table 1), the ratio of Gram‐positive to Gram‐negative PLFAs was increased by drought under *Holcus* but decreased under *Rumex* (species × treatment interaction *F*_1,27_ = 12.1, *P *=* *0.002; Fig. 3).

**Table 1:** Treatment means and SE (nmol g^−1^) and ANOVA table for microbial phospholipid fatty acid (PLFA) data.

Harvest	Species	Treatment	Total PLFA		Fungal PLFA		Bacterial PLFA		Gram^+^ PLFA		Gram^−^ PLFA	
			Mean	SE	Mean	SE	Mean	SE	Mean	SE	Mean	SE
End of drought	*Holcus*	Control	261.1	± 14.4	4.44	± 0.36	186.8	± 10.9	56.2	± 3.5	120.0	± 6.8
		Drought	215.2	± 16.6	3.12	± 0.23	155.4	± 12.1	111.5	± 5.9	236.3	± 29.6
	*Rumex*	Control	545.4	± 46.3	15.73	± 0.78	370.6	± 36.2	48.8	± 3.3	97.8	± 7.9
		Drought	498.8	± 36.3	15.19	± 1.10	338.8	± 24.0	93.7	± 6.8	224.3	± 15.8
2‐wk recovery	*Holcus*	Control	289.3	± 16.8	5.08	± 0.43	206.4	± 11.8	61.4	± 2.9	133.4	± 8.4
		Drought	240.9	± 20.3	3.9	± 0.27	173.3	± 14.3	112.0	± 4.2	257.4	± 12.9
	*Rumex*	Control	575.9	± 26.7	15.57	± 0.86	389.8	± 15.5	54.0	± 3.9	108.5	± 9.7
		Drought	541.1	± 19.3	12.51	± 0.51	370.3	± 12.5	103.8	± 3.2	244.4	± 9.0

			*F*_1,28_	*P*	*F*_1,28_	*P*	*F*_1,28_	*P*	*F*_1,28_	*P*	*F*_1,28_	*P*
Species			272	**< 0.0001**	523	**< 0.0001**	227	**< 0.0001**	263	**< 0.0001**	184	**< 0.0001**
Treatment			8.5	**0.01**	10.5	**0**	7.2	**0.01**	10.4	**0.003**	4.0	0.06
Harvest			3.9	0.06	0.5	0.47	3.8	0.06	3.5	0.07	3.1	0.09
Species × treatment			1.9	0.18	0.3	0.57	1.7	0.20	0.6	0.43	0.4	0.53
Species × harvest			0.07	0.79	5.5	**0.03**	0.04	0.85	0.02	0.89	0.3	0.61
Treatment × harvest			0.01	**0.01**	1.6	0.21	0.02	0.89	0.5	0.50	0.02	0.88
Species × treatment × harvest			0	0.96	2.2	0.15	0.02	0.94	0.5	0.49	0.0	0.99

Bold text indicates statistical significance.

### Interactive effects of drought and species identity on soil inorganic N and DOC concentrations and on root exudation rates

Soil nitrate and ammonium concentrations showed a similar pattern, but nitrate concentrations in particular were much higher under *Holcus* than under *Rumex* (Table 2). Both nitrate and ammonium were increased by drought, but ammonium concentrations only increased under *Holcus*; these increases persisted until the 2 wk recovery harvest. Total dissolved N concentrations followed the pattern of plant‐available nitrate (Table 2). Similar to inorganic N concentrations, soil DOC concentrations were higher under *Holcus* than under *Rumex*. However, in contrast to inorganic N, at the final sampling DOC was reduced under *Holcus* but increased under *Rumex*.

**Table 2:** Treatment means and SE (μg g^−1^) and ANOVA table for soil dissolved carbon and nitrogen pools.

Harvest	Species	Treatment	DOC		Nitrate		Ammonium		Total dissolved nitrogen	
			Mean	SE	Mean	SE	Mean	SE	Mean	SE
End of drought	*Holcus*	Control	52	± 3.5	103.6	± 37.2	6.4	± 0.5	66.8	± 21.4
		Drought	70.4	± 6.1	174.5	± 1.1	21.1	± 1.6	145.4	± 32.3
	*Rumex*	Control	32.8	± 4.4	5.6	± 0.7	5.1	± 0.2	2.9	± 1.9
		Drought	32.7	± 3.6	11.4	± 3.2	6.4	± 0.7	14	± 2.1
2‐wk recovery	*Holcus*	Control	59.2	± 4.4	115.6	± 37.1	6	± 0.9	98.3	± 28.2
		Drought	49.5	± 2.6	273.9	± 56.3	10.9	± 1.0	159.1	± 32.8
	*Rumex*	Control	37.3	± 1.8	5.8	± 0.4	4.1	± 0.5	1.1	± 1.1
		Drought	47.5	± 3.4	7.5	± 1.0	4.9	± 0.4	18.4	± 1.3

			*F*_1,28_	*P*	*F*_1,28_	*P*	*F*_1,28_	*P*	*F*_1,28_	*P*
Species			51.8	**< 0.0001**	101.4	**< 0.0001**	80.3	**< 0.0001**	49.8	**< 0.0001**
Treatment			2.8	0.1	7.7	**0.01**	60.3	**< 0.0001**	28.1	**< 0.0001**
Harvest			0.25	0.62	0	0.99	18.9	**0.0002**	0.2	0.68
Species × treatment			0.013	0.91	1.7	0.2	24.3	**< 0.0001**	17.6	**0.0002**
Species × harvest			8.6	**0.007**	0.2	0.64	1	0.32	0.4	0.54
Treatment × harvest			2.5	0.12	0	0.94	3.9	0.06	0.4	0.53
Species × treatment × harvest			11.6	**0.002**	0.2	0.61	3.5	0.07	0.7	0.43

DOC, dissolved organic carbon

Bold text indicates statistical significance.

**Figure 4:**
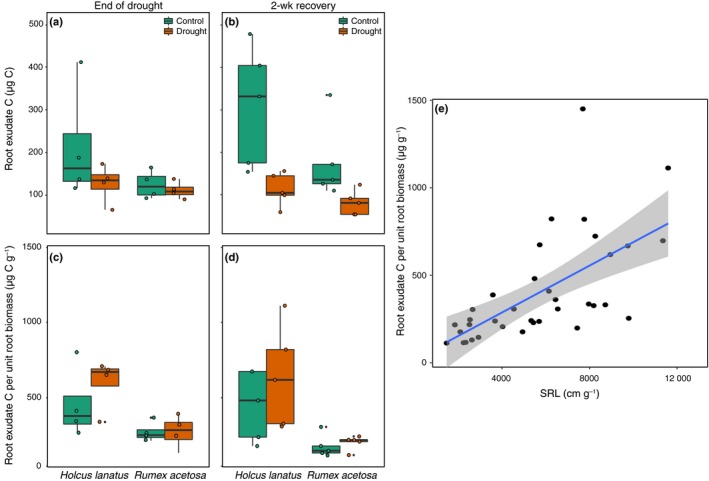
(a, b) Total root‐exuded carbon (C), (c, d) root‐exuded C per unit root biomass, and (e) the positive relationship between root exudate C per unit root biomass and specific root length (SRL) for end of drought and 2‐wk recovery harvests as affected by species identity (*Holcus lanatus* and *Rumex acetosa*) and drought. Lines in boxes (a–d) represent median, top and bottom of boxes represent first and third quartiles, and whiskers represent 1.5 interquartile range; data points represent single observations. In (e), line and shading represent linear regression and 95% confidence interval. See the Results section for statistics.

The absolute amount of root exudate C collected was higher from *Holcus* than from *Rumex* (main effect of species *F*_1,24_ = 6.5, *P *=* *0.017; Fig. 4), and was reduced by drought in both species, particularly at the final harvest (main effect of drought *F*_1,24_ = 14.5, *P *=* *0.0009; Fig. 4). However, when expressed per unit of root biomass, this pattern disappeared, and, while highly variable, *Holcus* had higher root exudation rates per unit root biomass (main effect of species *F*_1,24_ = 19.7, *P *=* *0.0002; Fig. 4). Root exudation per unit root biomass was positively related to SRL (*R* ^2^ = 0.39, *P *<* *0.0001; Fig. 4e). In addition, we found that whereas the total amount of C exuded was not related to soil nitrate concentrations and microbial biomass C, the amount of C exuded per unit root biomass was positively related to soil nitrate and negatively related to microbial biomass C (*P *<* *0.0001, *R* ^2^ = 0.48, and *P *=* *0.0002, *R* ^2^ = 0.34; Fig. S2).

### Effects of soil species conditioning, soil drought treatment, root exudate species identity, and root exudate drought treatment on root‐exudate‐induced respiration

At the end of drought harvest, the amount of root exudate C added to soil did not predict water‐normalized CO_2_ production rates (*t* = 0.176, *P *=* *0.86; Fig. S3a). There were no significant effects of soil species conditioning, soil drought treatment, root exudate species, and root exudate drought treatment on root exudate C‐standardized CO_2_ production (Table S1). However, when expressed per unit soil microbial biomass C, *Holcus* soils respired more C than *Rumex* soils did (Satterthwaite's *F*_1,7.9_ = 31.4, *P *=* *0.0005; Fig. S4a), and droughted *Holcus* exduates only increased C respired in control soil (Satterthwaite's *F*_1,190.9_ = 7.44, *P *=* *0.006; Fig. S4b,c).

**Figure 5:**
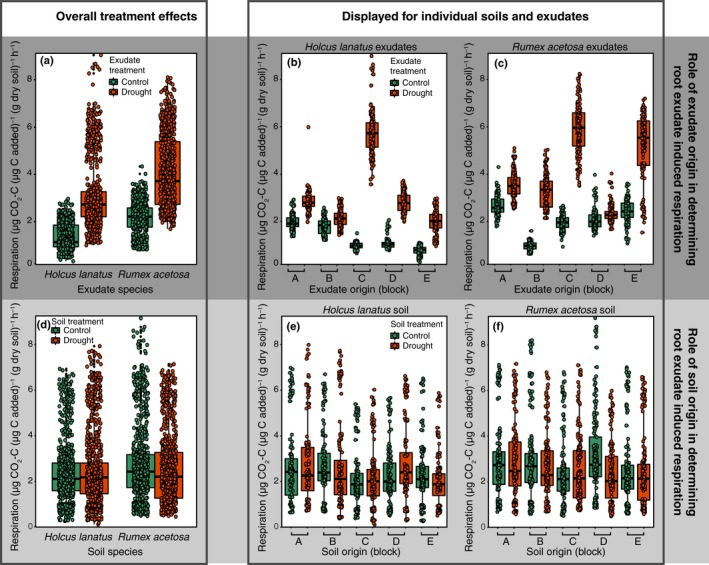
Root‐exudate‐induced respiration as affected by (a–c) exudate treatment (i.e. drought or control history) and (d–f) soil treatment (i.e. drought or control history) at the 2 wk recovery harvest of *Holcus lanatus* and *Rumex acetosa*. Overall treatment effects (a, d) as well as the effect of individual root exudate combinations (b, c), and individual soil combinations (e, f) are displayed. (a–c) and (d–f) represent the same data but split for exudate treatment and soil treatment, respectively. Lines in boxes represent median, top and bottom of boxes represent first and third quartiles, and whiskers represent 1.5 interquartile range; data points represent single observations. Uppercase letters on *x*‐axes represent experimental blocks in which the pots were organized, with square brackets indicating control–drought pairs within blocks. See the Results section for statistics.

At the final harvest after 2 wk of recovery of plant–soil systems, water‐normalized root‐exudate‐induced respiration showed a positive relationship with root exudate C added (*t* = 2.73, *P *=* *0.016; Fig. S3b). Furthermore, and in contrast with the end‐of‐drought harvest, there were clear effects of root exudate drought treatment and root exudate species on C‐standardized CO_2_ production. Importantly, droughted root exudates strongly increased specific respiration rates across both species and all soil treatments (Satterthwaite's *F*_1,15.34_ = 19.0, *P *=* *0.0005; Fig. 5; see Table S2 for the full model output). In other words, even after having recovered from drought for 2 wk, root exudates from droughted plants accelerated soil respiration rates irrespective of which species had been grown in soils or whether soils had a drought history. In addition, overall, root exudates collected from *Rumex* triggered higher respiration rates per unit root exudate C added (Satterthwaite's *F*_1,15.34_ = 5.3, *P *=* *0.036; Fig. 5). Specifically, *Rumex* exudates tended to increase specific respiration rates most in *Holcus* soils with a drought history (soil species × soil treatment × exudate species interaction Satterthwaite's *F*_1,324.17_ = 3.2, *P *=* *0.077; Fig. S5).

When root‐exudate‐induced respiration was expressed per unit microbial biomass C, a similar, but less strong, pattern appeared, with droughted exudates inducing higher respiration rates, and *Rumex* exudates triggering more respiration than *Holcus* exudates did (Satterthwaite's *F*_1,12.1_ = 17.7, *P *=* *0.001, and Satterthwaite's *F*_1,12.1_ = 5.0, *P *=* *0.04, respectively; Fig. S6). In addition, and similar to the end‐of‐drought harvest, *Holcus* soils showed higher root exudate induced respiration per unit microbial biomass (Satterthwaite's *F*_1,17.6_ = 9.6, *P *=* *0.006; Fig. S6).

### Drought and species‐specific effects on ecosystem respiration and the contribution of root‐exudate‐induced respiration

**Figure 6:**
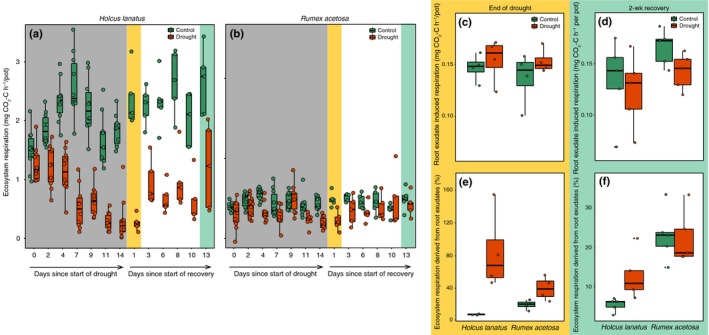
Ecosystem respiration for (a) *Holcus lanatus* and (b) *Rumex acetosa* over time as affected by drought, and (c, d) the total amount of root‐exudate‐induced respiration and (e, f) the percentage of root‐exudate‐induced respiration of total ecosystem respiration for the two harvests as affected by species identity and drought. In (a, b), grey shading indicates the duration of the drought, and in all figures the yellow shading indicates the end‐of‐drought harvest and green shading indicates the 2‐wk recovery harvest. Lines in boxes represent median, top and bottom of boxes represent first and third quartiles, and whiskers represent 1.5 interquartile range; data pointts represent single observations. See the Results section for statistics.

Overall, ecosystem respiration was much higher for *Holcus* than for *Rumex* (repeated‐measures ANOVA main effect for species *F*_1,383_ = 470, *P *<* *0.0001; Fig. 6a,b). Drought reduced respiration rates more for *Holcus* than it did for *Rumex*, and respiration rates in plant–soil systems with *Holcus* had not yet recovered from drought at the final harvest (species × treatment × date interaction *F*_1,383_ = 15.7, *P *=* *0.0001; Fig. 6a,b). Total root‐exudate‐induced respiration did not differ between species and drought treatments at either harvest, but was slightly higher in plant–soil systems with *Rumex* at the final harvest (main effect of species *F*_1,12_ = 5.7, *P *=* *0.035; Fig. 6c,d). As a result, at the end of the drought, root‐exudate‐induced respiration contributed much more to total ecosystem respiration in drought treatments (main effect of treatment *F*_1,9_ = 14.6, *P *=* *0.004; Fig. 6e), whereas, at the final harvest, *Rumex* root exudates contributed more to ecosystem respiration than *Holcus* exudates did (main effect of species *F*_1,12_ = 28.8, *P *=* *0.0002; Fig. 6f). RNC was the best predictor of the average root‐exudate‐induced respiration for individual root exudates (*R* ^2^ = 0.29, *P *=* *0.015; Fig. S7).

## Discussion

Using a unique experimental approach, we show that, across two plant species with widely different growth strategies and root systems, drought‐induced changes in root exudation have clear functional implications. Despite a 2 wk recovery period, exudates from droughted plants induced higher soil respiration rates than exudates from control plants did. These findings are in line with previous studies that suggest plants adjust rates of root exudation to overcome environmental challenges (Hamilton & Frank, 2001; Phillips *et al*., 2011). But here, we show that changes in root exudates other than their quantity are responsible for the effect on functioning; our findings suggest that plants change the composition of their root exudates to increase microbial activity after drought, which may in turn facilitate plant regrowth through increased rates of nutrient mineralization.

Drought strongly affected soil microbial communities and soil C and N availability, but these changes only affected root‐induced respiration when expressed per unit of microbial biomass. Though lower in biomass, microbial communities in *Holcus* soils released more C in response to root exudate addition than those in *Rumex* soils did. This suggests that microbial communities in *Holcus* soils are limited by labile C, which is supported by high dissolved N concentrations in these soils. Whereas soil DOC concentrations and root exudation were also higher in *Holcus* soils, supporting earlier findings (Ladygina & Hedlund, 2010), the higher ratio of Gram‐positive to Gram‐negative bacteria might indicate that *Holcus* microbial communities were limited by labile C (Fanin *et al*., 2019). By contrast, root exudate origin strongly determined root‐exudate‐induced respiration; and strikingly, within pairs (blocks), exudates from droughted plants almost always induced more respiration than those from control plants did. Although we did not identify qualitative changes in root exudates in response to drought, we suggest three potential mechanisms underlying the increase in respiration per unit root exudate C. First, drought has been shown to increase carbohydrate storage in roots (Karlowsky *et al*., 2018a,b) and thus might also increase the content of simple sugars in root exudates, which, as a non‐specific, easily accessible resource, stimulates the entire active microbial community during recovery from drought (Landi *et al*., 2006; Karlowsky *et al*., 2018a). Second, drought has been shown to increase the concentration of secondary metabolites in root exudates (Gargallo‐Garriga *et al*., 2018), which might stimulate specific microbial functional groups and reduce others, which could potentially result in increased N availability for plant uptake (Liu *et al*., 2015; Czaban *et al*., 2018). Third, plants might increase the exudation of organic acids to mobilize inorganic P and thus increase plant P uptake during regrowth (Canarini *et al*., 2019); several studies show that organic acids have the potential to prime the decomposition of soil organic C (Falchini *et al*., 2003; Brant *et al*., 2006).

Strikingly, the total respiration derived from root exudates was similar under control and drought conditions, suggesting that the effect on respiration of the change in root exudate quality compensated for the reduction in root exudate quantity. This finding supports our hypothesis that we have identified a controlled mechanism to maintain microbial activity during drought recovery. Moreover, while root‐exudate‐induced respiration was highly variable across exudates from individuals, almost all exudates caused an apparent priming effect; that is, a higher amount of respired C than the amount of C added (Blagodatskaya & Kuzyakov, 2008). This is in line with findings that root exudates, or their major components, can cause priming of soil C (Falchini *et al*., 2003; Landi *et al*., 2006; Zhu *et al*., 2014; Shahzad *et al*., 2015), in particular under changing environmental conditions such as elevated CO_2_ or warming (Bengtson *et al*., 2012; Dijkstra *et al*., 2013; Perveen *et al*., 2014). Importantly, our data suggest that, despite their lower concentrations, root exudates from droughted plants might particularly increase the loss of soil C. Thus, we identify a novel mechanism through which drought can continue to affect ecosystem C cycling. Though it is well known that dry–wet cycles can result in a flush of CO_2_ release from soil, the origin of which is hotly debated (e.g. Schimel, 2018), we show here that changes in root exudates might contribute to the loss of soil C after droughted soils are rewetted.

Ecosystem respiration rates were much higher for *Holcus*, and were also more reduced by drought for *Holcus* than for *Rumex*. Moreover, *Holcus* ecosystem respiration had still not recovered to control levels at the 2 wk recovery harvest. Strong species‐specific responses of soil respiration to drought have been shown before (Yan *et al*., 2017), and depressed soil respiration rates as a result of drought legacy have been attributed to reduced belowground C input (Goransson *et al*., 2013). However, our data show that, despite lower root exudation under drought, total root‐exudate‐induced respiration was not affected by drought. As a result, the percentage ecosystem respiration induced by root exudates was significantly increased under drought for *Holcus* (Fig. 5). This is an important finding that is in contrast with ^13^C‐labelling studies that show reduced root‐exudate‐derived respiration under drought (Ruehr *et al*., 2009; Fuchslueger *et al*., 2014) but which do not measure additional soil C release triggered by root exudates, as we have done here. The proportion of root exudate respiration of total ecosystem respiration we found is in line with estimates of root exudation rates (5–30%; Fig. 5f) (Jones *et al*., 2004, 2009), confirming that the respiration rates we have measured are realistic. Notably, consistent with respiration responses to warming (Wang *et al*., 2014), our findings also show that the rate of root exudation, the respiration triggered by these exudates, and total ecosystem respiration are decoupled under drought and recovery.

There is a lot of interest in root processes and how they modify ecosystem response to global change (Bardgett *et al*., 2014), and root biomass as well as root traits are increasingly used as a predictor for changes in belowground functioning under drought (De Vries *et al*., 2016; Bloor *et al*., 2018). We found that whereas drought reduced root biomass and rates of root exudation across two species, these did not result in a decrease of root‐exudate‐induced respiration. In addition, we found that root exudation per unit root biomass increased with SRL – which was also found in a recent study by Guyonnet *et al*. (2018)), although they did not express root exudation per unit root biomass – but root exudation only weakly predicted root‐exudate‐induced respiration at the 2 wk recovery harvest (Fig. S3). The best predictor for root‐exudate‐induced respiration was RNC, which is in line with findings from Zhou *et al*. (2018), who found that root respiration increased with higher RNC, which in itself was indicative for a shorter root lifespan. We found that drought increased RNC, but these results should be viewed with caution as this increase might be a result of reduced plant growth under drought, and younger roots have higher RNC (De Vries *et al*., 2016). Moreover, the increase in root‐exudation‐induced respiration we find here with droughted root exudates might also be caused by a delay in plant development, as it is well known that root exudation patterns change significantly during plant development (Aulakh *et al*., 2001). Thus, our findings indicate that caution is needed in using changes in root biomass and root traits to predict the response of root processes to drought, and they highlight the need for more work to elucidate the functional role of changes in the quantity and quality of root exudation in response to changing environmental conditions and across different species. The fact that both of our species are fast growing poses the question whether the same pattern can be detected in slow‐growing species, which have fundamentally different root exudation strategies (Kastovska *et al*., 2015).

We have presented a novel method of collecting root exudates and using these exudates in the well‐described MicroResp system (Campbell *et al*., 2003). This set‐up allows for disentangling the implications of differences in the quantity and quality of root exudates, as well as differences in soil properties and microbial communities, for respiration, which is not possible using ^13^CO_2_ pulse labelling. However, root exudates collected and readded in aqueous solution to isolated soil samples might well behave differently than in an undisturbed rhizosphere (Oburger *et al*., 2013; Oburger & Jones, 2018). Ideally, our method would be combined with ^13^CO_2_ pulse labelling and flux measurements in an intact system, as well as detailed analysis of ^13^C‐enriched root exudate metabolites and microbial communities.

Our findings have important implications for understanding plant and ecosystem response to drought, and they show that reduced root exudation rates in response to drought do not result in lower root‐exudate‐induced respiration rates. Moreover, we show that root exudates from droughted plants can potentially continue to affect ecosystem C cycling through the decomposition of soil C. We found this pattern across two distinct species, which supports our hypothesis that we have identified an evolutionary strategy to overcome adverse environmental conditions through the stimulation of microbial activity and the subsequent mineralization of nutrients.

## Author contributions

FTdV and ALS conceived and designed the experiment; ALS, FS, RW, RM and HL performed the experiments and laboratory analyses; FTdV, ALS, HL and AW analysed and interpreted the data; FTdV wrote the manuscript with contributions from all authors.

## Supporting information

Please note: Wiley Blackwell are not responsible for the content or functionality of any Supporting Information supplied by the authors. Any queries (other than missing material) should be directed to the *New Phytologist* Central Office.

**Fig. S1** Percentage of soil water holding capacity (WHC) in the different experimental treatments over time.
**Fig. S2** Relationship between total root exudate C and root exudate C per unit root with soil nitrate concentration and microbial biomass C.
**Fig. S3** Relationship of water normalised root exudate induced respiration and the amount of C added.
**Fig. S4** Root exudate induced respiration per unit C added expressed per unit soil microbial biomass C, as affected by soil species and root exudate treatment and species at the end of drought harvest.
**Fig. S5** Root exudate induced respiration for *Holcus lanatus* and *Rumex acetosa* soil at the 2‐wk recovery harvest as affected by soil treatment and exudate species.
**Fig. S6** Root exudate induced respiration expressed per unit soil microbial biomass, as affected by exudate species and treatment and soil species and treatment at the 2‐wk recovery harvest.
**Fig. S7** Relationship between root exudate respiration averaged per individual root exudate (*i.e*. derived from an individual plant) and RNC of that individual plant.
**Table S1** Model output for C standardised CO_2_ production at the end of drought harvest.
**Table S2** Model output for C standardised CO_2_ production at the 2‐wk recovery harvest.
