# Supplementary material for: Changes in root‐exudate‐induced respiration reveal a novel mechanism through which drought affects ecosystem carbon cycling
Source: New Phytol. 2019 Jul 24;224(1):132–45. doi: 10.1111/nph.16001 (PMC6771481; doi:10.1111/nph.16001)
Supplement: Supplementary file 1 — Fig. S1 Percentage of soil water holding capacity (WHC) in the different experimental treatments over time. Fig. S2 Relationship between total root exudate C and root exudate C per unit root with soil nitrate concentration and microbial biomass C. Fig. S3 Relationship of water normalised root exudate induced respiration and the amount of C added. Fig. S4 Root exudate induced respiration per unit C added expressed per unit soil microbial biomass C, as affected by soil species and root exudate treatment and species at the end of drought harvest. Fig. S5 Root exudate induced respiration for Holcus lanatus and Rumex acetosa soil at the 2‐wk recovery harvest as affected by soil treatment and exudate species. Fig. S6 Root exudate induced respiration expressed per unit soil microbial biomass, as affected by exudate species and treatment and soil species and treatment at the 2‐wk recovery harvest. Fig. S7 Relationship between root exudate respiration averaged per individual root exudate (i.e. derived from an individual plant) and RNC of that individual plant. Table S1 Model output for C standardised CO2 production at the end of drought harvest. Table S2 Model output for C standardised CO2 production at the 2‐wk recovery harvest. [file NPH-224-132-s001.pdf]

***New Phytologist* Supporting Information**

Article title: Changes in root exudate induced respiration reveal a novel mechanism through which drought affects ecosystem carbon cycling
Authors: Franciska T. de Vries, Alex Williams, Fiona Stringer, Robert Willcocks, Rosie McEwing, Holly Langridge, and Angela L. Straathof
Article acceptance date: 02 June 2019

The following Supporting Information is available for this article:

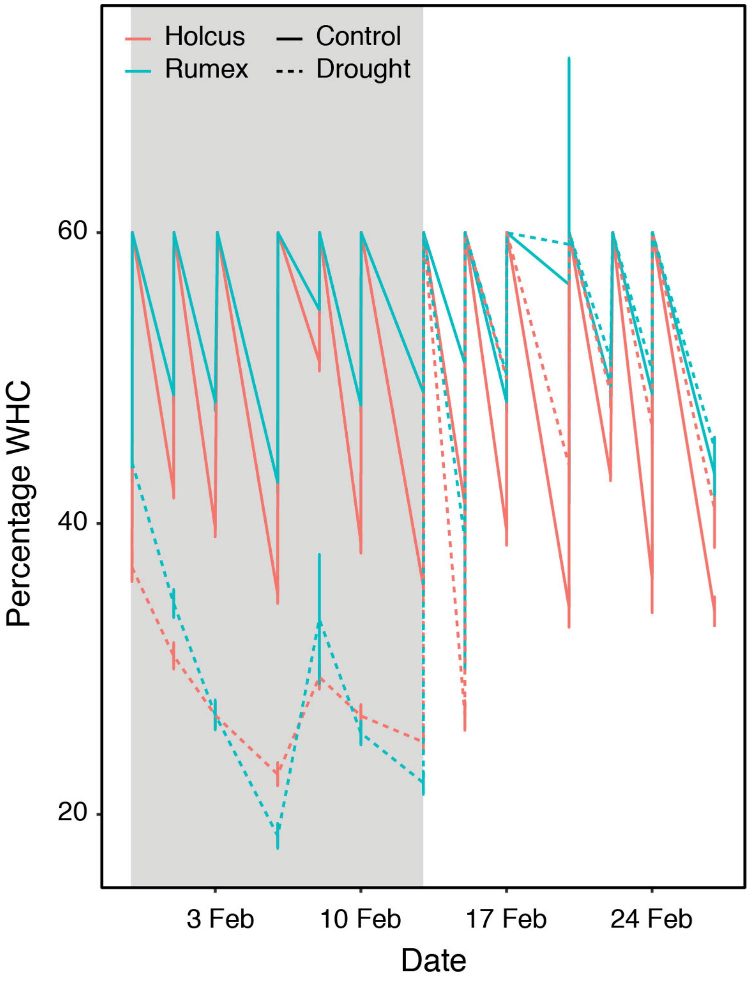

Fig. S1 Percentage of soil water holding capacity (WHC) in the different experimental treatments over time. Grey shading indicates the duration of the experimental drought. Lines connect mean values (n = 4), bars represent 1 SE.

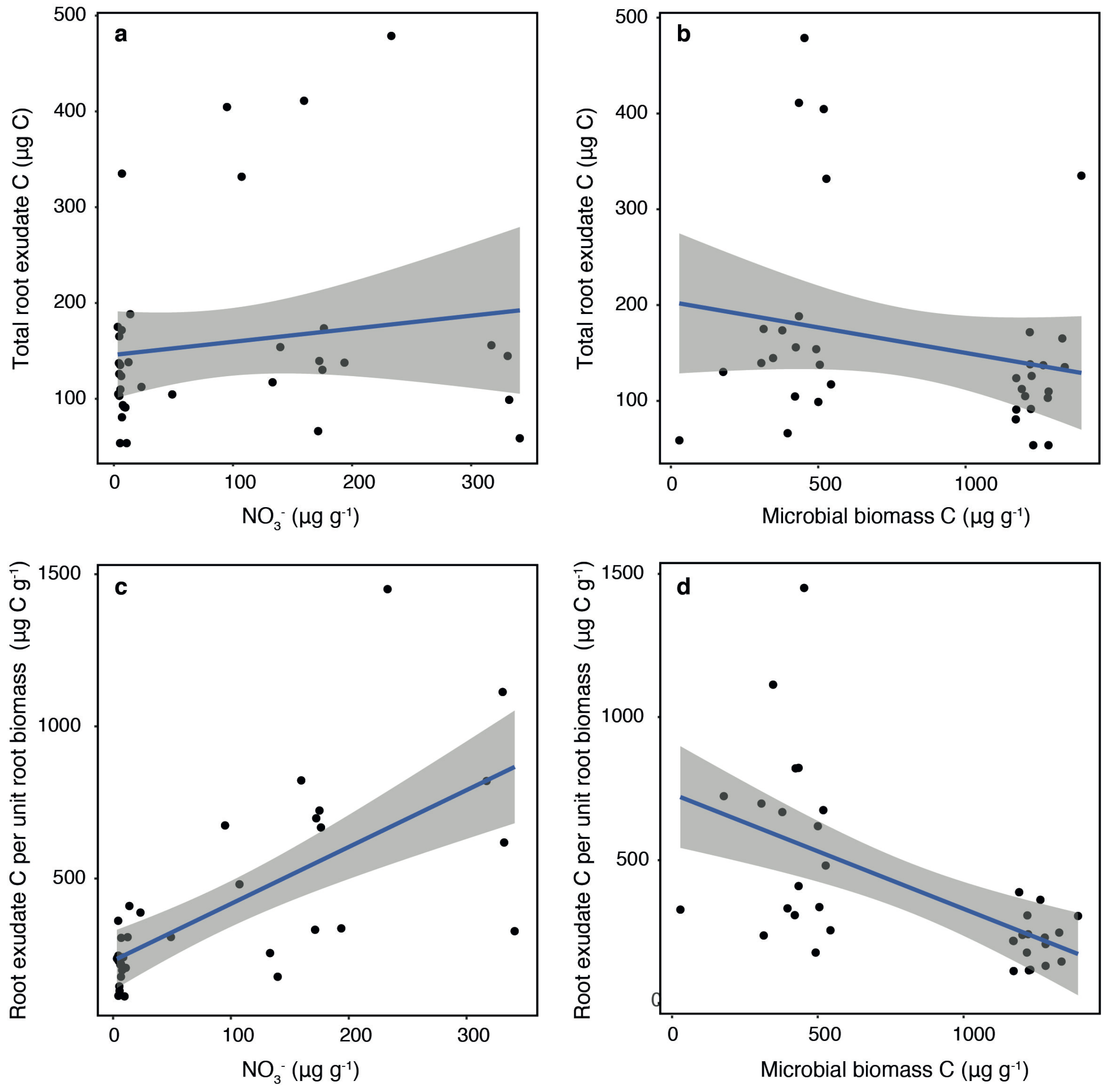

Fig. S2 Relationship between total root exudate C (a, b) and root exudate C per unit root biomass (c, d) with soil nitrate concentration and microbial biomass C. Line and shading represent linear regression and 95% confidence interval.

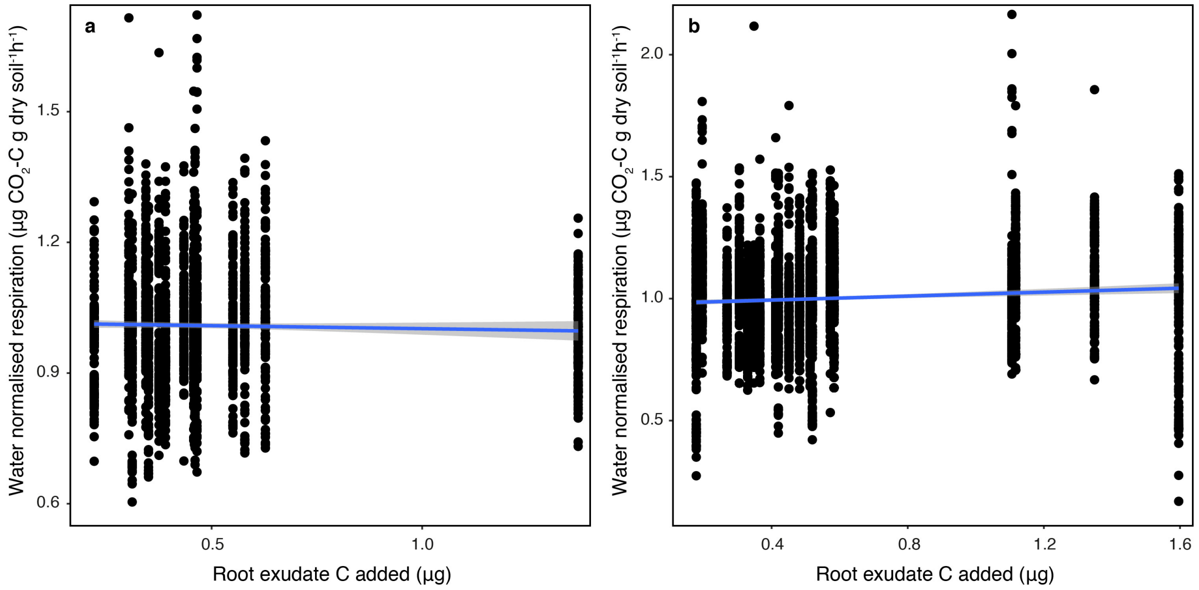

Fig. S3 Relationship of water normalised root exudate induced respiration and the amount of C added for the End of drought harvest (a) and the 2-week-recovery harvest (b). See main text for statistics. Lines and shading represent linear regression and 95% confidence interval.

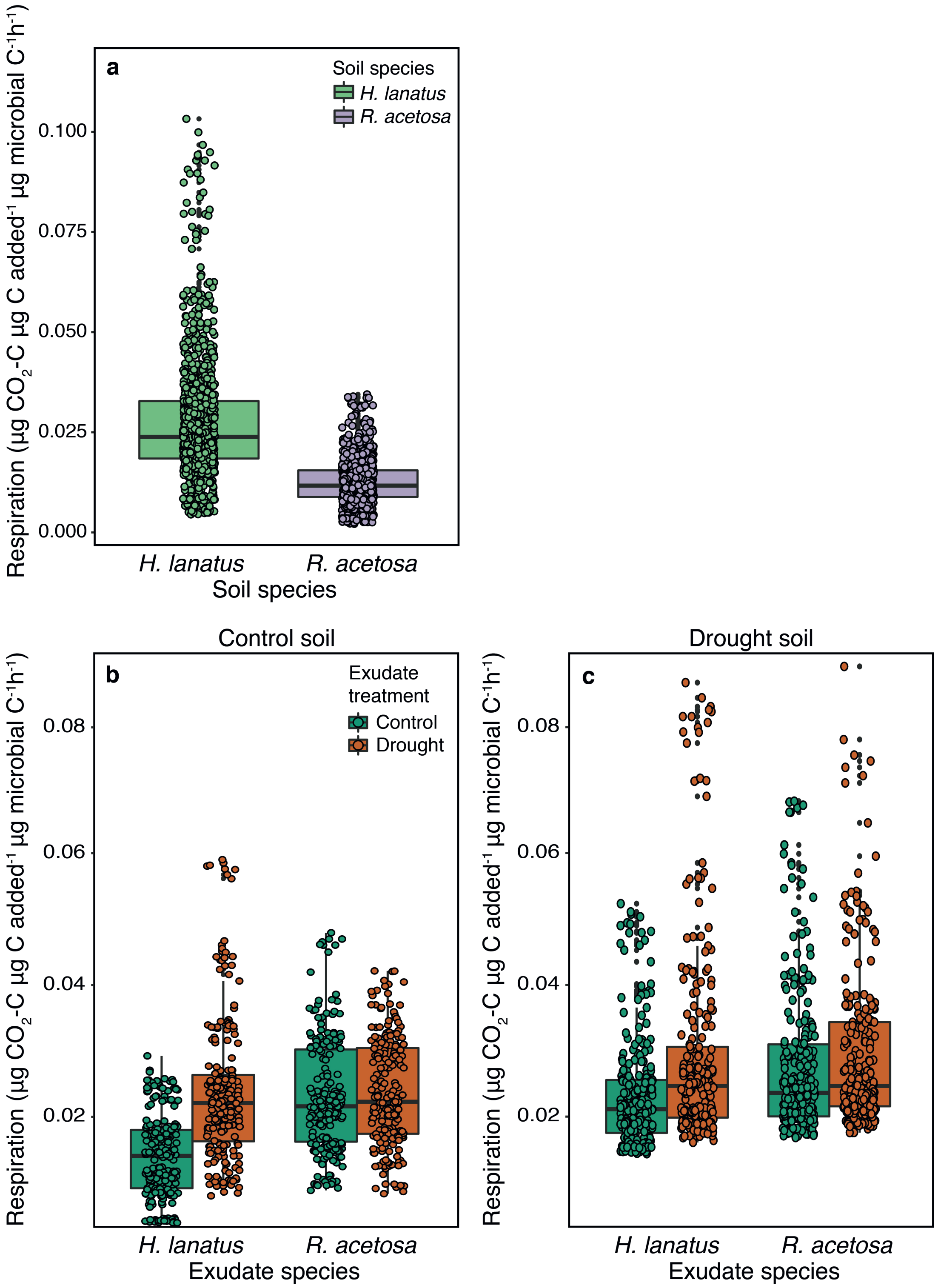

Fig. S4 Root exudate induced respiration per unit C added expressed per unit soil microbial biomass C, as affected by soil species (a) and root exudate treatment and species (b, c) at the end of drought harvest. Lines and shading represent linear regression and 95% confidence interval. See Results section for statistics.

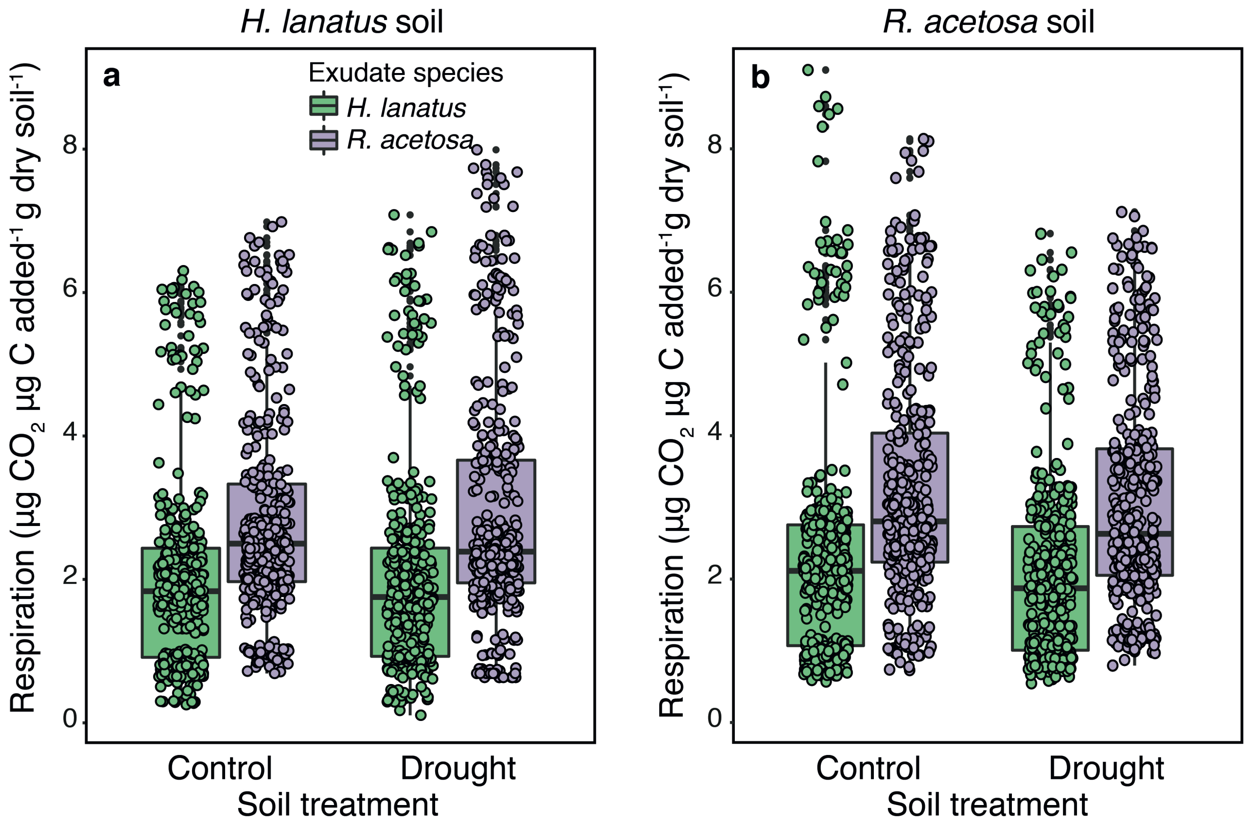

Fig. S5 Root exudate induced respiration for *Holcus* (a) and *Rumex* (b) soil at the 2-week recovery harvest as affected by soil treatment and exudate species. Lines in boxes represent median, top and bottom of boxes represent first and third quartiles, and whiskers represent 1.5 interquartile range; dots represent single observations. See Results section for statistics.

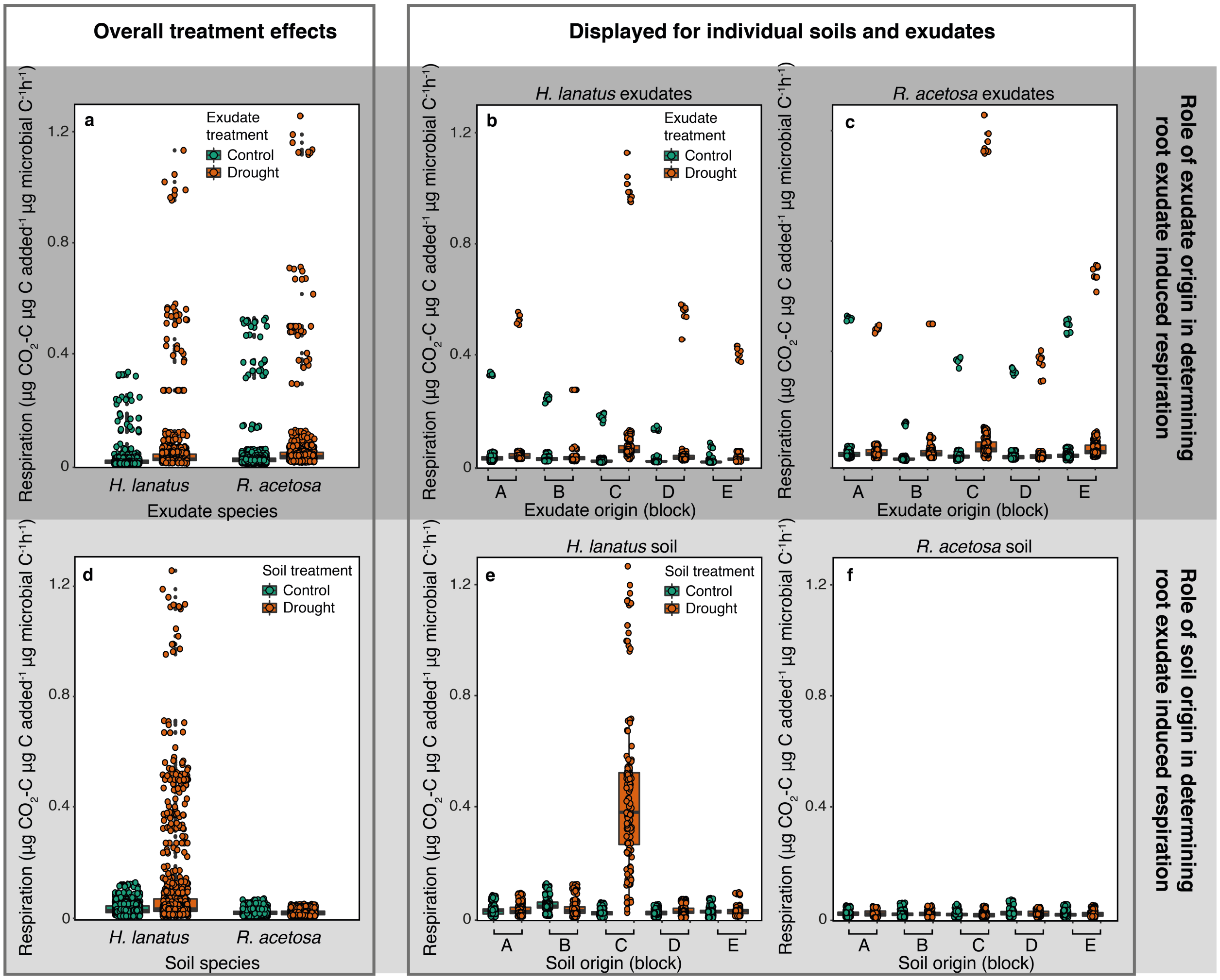

Fig. S6 Root exudate induced respiration expressed per unit soil microbial biomass, as affected by exudate species and treatment (a, b, c) and soil species and treatment (d, e, f) at the 2-week recovery harvest. See main text for statistics. Lines in boxes represent median, top and bottom of boxes represent first and third quartiles, and whiskers represent 1.5 interquartile range; dots represent single observations. See Results section for statistics.

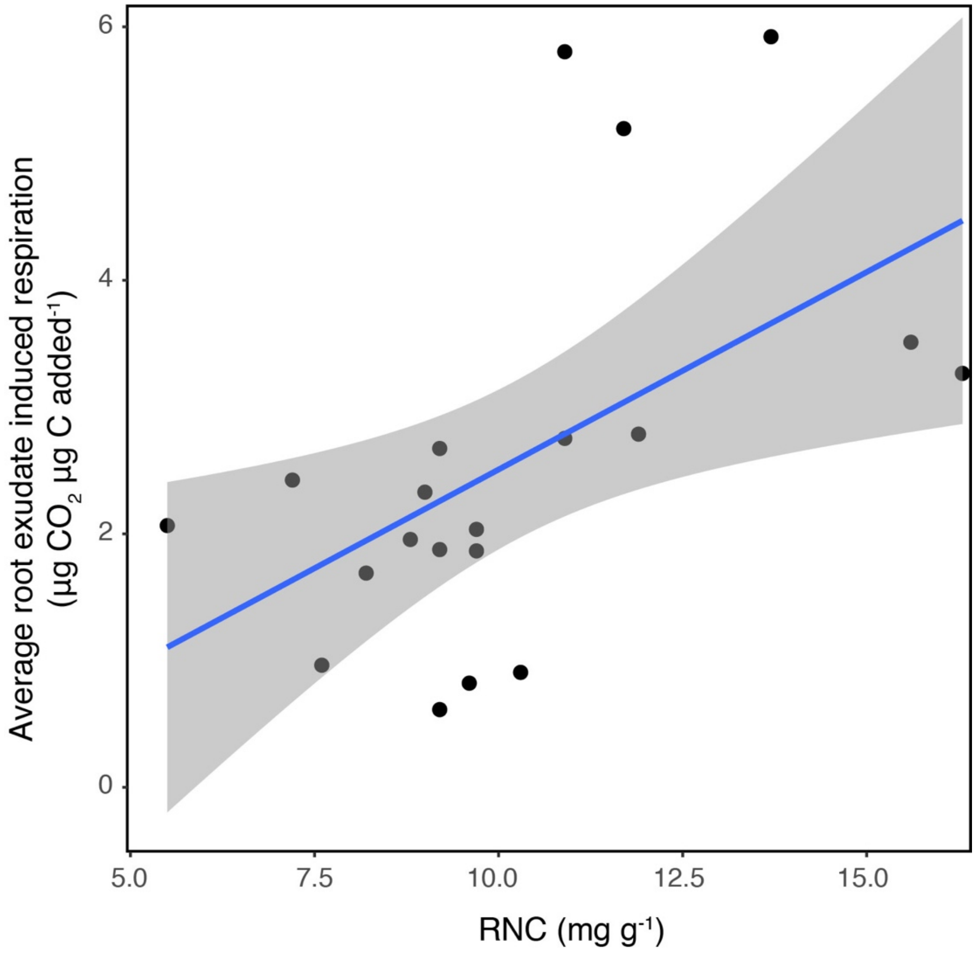

Fig. S7 Relationship between root exudate respiration averaged per individual root exudate (*i.e.* derived from an individual plant) and RNC (root nitrogen content) of that individual plant. Line and shading represent linear regression and 95% confidence interval.

Table S1 Model output for C standardised CO_2 production at the end of drought harvest.

```
> lm2<-lmer(log(CO2_C6) ~ Soil_Species*Soil_Trtmt*Exud_Species*Exud_Trtmt + (1|Soil_block/Soil_id/S.E_combo) + (1|exud_block/exud_id) +
(1|Plate/Row), data=h1, na.action=na.omit)
> summary(lm2)
Linear mixed model fit by REML. t-tests use Satterthwaite's method ['lmerModLmerTest']
Formula: log(CO2_C6) ~ Soil_Species * Soil_Trtmt * Exud_Species * Exud_Trtmt +      (1 | Soil_block/Soil_id/S.E_combo) + (1 |
exud_block/exud_id) +      (1 | Plate/Row)
   Data: h1

REML criterion at convergence: -4852.2

Scaled residuals:
    Min      1Q  Median      3Q     Max
-5.4670 -0.4903 -0.0196  0.4770  5.1521

Random effects:
 Groups                         Name        Variance  Std.Dev.
 S.E_combo:(Soil_id:Soil_block) (Intercept) 5.914e-03 0.076902
 Row:Plate                      (Intercept) 1.747e-03 0.041802
 Plate                          (Intercept) 1.814e-03 0.042588
 exud_id:exud_block             (Intercept) 1.406e-01 0.374950
 Soil_id:Soil_block             (Intercept) 3.729e-03 0.061067
 exud_block                     (Intercept) 9.107e-05 0.009543
 Soil_block                     (Intercept) 5.027e-03 0.070902
 Residual                                   2.887e-03 0.053727
Number of obs: 2048, groups:  S.E_combo:(Soil_id:Soil_block), 256; Row:Plate, 224; Plate, 28; exud_id:exud_block, 16; Soil_id:Soil_block, 16;
exud_block, 4; Soil_block, 4

Fixed effects:
                                                                         Estimate Std. Error         df t value Pr(>|t|)
(Intercept)                                                              0.446515   0.194589  13.683684   2.295   0.0381 *
Soil_SpeciesRumex                                                        0.020960   0.052238  15.447640   0.401   0.6937
Soil_TrtmtDrought                                                        0.025589   0.052372  15.599241   0.489   0.6319
Exud_SpeciesRumex                                                        0.469453   0.266694  11.886390   1.760   0.1040
Exud_TrtmtDrought                                                        0.479507   0.266755  11.897423   1.798   0.0977 .
Soil_SpeciesRumex:Soil_TrtmtDrought                                     -0.050306   0.073649  15.259593  -0.683   0.5048
Soil_SpeciesRumex:Exud_SpeciesRumex                                     -0.030573   0.040670 199.063803  -0.752   0.4531
Soil_TrtmtDrought:Exud_SpeciesRumex                                     -0.011811   0.041302 203.972220  -0.286   0.7752
Soil_SpeciesRumex:Exud_TrtmtDrought                                     -0.030190   0.042047 207.944245  -0.718   0.4736
Soil_TrtmtDrought:Exud_TrtmtDrought                                      0.002852   0.041371 203.672565   0.069   0.9451
Exud_SpeciesRumex:Exud_TrtmtDrought                                     -0.409242   0.377141  11.883782  -1.085   0.2994
Soil_SpeciesRumex:Soil_TrtmtDrought:Exud_SpeciesRumex                    0.005830   0.058237 202.775791   0.100   0.9204
Soil_SpeciesRumex:Soil_TrtmtDrought:Exud_TrtmtDrought                   -0.001991   0.058926 206.024553  -0.034   0.9731
Soil_SpeciesRumex:Exud_SpeciesRumex:Exud_TrtmtDrought                    0.020279   0.058313 203.031809   0.348   0.7284
Soil_TrtmtDrought:Exud_SpeciesRumex:Exud_TrtmtDrought                    0.034744   0.058015 201.680735   0.599   0.5499
Soil_SpeciesRumex:Soil_TrtmtDrought:Exud_SpeciesRumex:Exud_TrtmtDrought  0.030442   0.083823 207.805428   0.363   0.7168
---
Signif. codes:  0 '***' 0.001 '**' 0.01 '*' 0.05 '.' 0.1 ' ' 1

Correlation matrix not shown by default, as p = 16 > 12.
Use print(x, correlation=TRUE)  or
    vcov(x)        if you need it


> plot(lm2)
> anova(lm2)
Type III Analysis of Variance Table with Satterthwaite's method
                                                 Sum Sq   Mean Sq NumDF   DenDF F value Pr(>F)
Soil_Species                                  0.0017014 0.0017014     1   8.988  0.5894 0.4623
Soil_Trtmt                                    0.0002461 0.0002461     1   8.980  0.0853 0.7769
Exud_Species                                  0.0056490 0.0056490     1  11.680  1.9570 0.1878
Exud_Trtmt                                    0.0063412 0.0063412     1  11.679  2.1968 0.1648
Soil_Species:Soil_Trtmt                       0.0011580 0.0011580     1   8.948  0.4012 0.5423
Soil_Species:Exud_Species                     0.0006681 0.0006681     1 202.484  0.2315 0.6310
Soil_Trtmt:Exud_Species                       0.0016924 0.0016924     1 208.288  0.5863 0.4447
Soil_Species:Exud_Trtmt                       0.0011493 0.0011493     1 210.367  0.3982 0.5287
Soil_Trtmt:Exud_Trtmt                         0.0047247 0.0047247     1 207.149  1.6368 0.2022
Exud_Species:Exud_Trtmt                       0.0028654 0.0028654     1  11.678  0.9927 0.3393
Soil_Species:Soil_Trtmt:Exud_Species          0.0007665 0.0007665     1 200.300  0.2655 0.6069
Soil_Species:Soil_Trtmt:Exud_Trtmt            0.0002966 0.0002966     1 203.331  0.1028 0.7489
Soil_Species:Exud_Species:Exud_Trtmt          0.0021961 0.0021961     1 199.185  0.7608 0.3841
Soil_Trtmt:Exud_Species:Exud_Trtmt            0.0043353 0.0043353     1 199.486  1.5019 0.2218
Soil_Species:Soil_Trtmt:Exud_Species:Exud_Trtmt 0.0003807 0.0003807     1 207.805  0.1319 0.7168
```

Table S2 Model output for C standardised CO_2 production at the 2-week recovery harvest.

```
> lm3<-lmer(log(CO2_C6) ~ Soil_Species*Soil_Trtmt*Exud_Species*Exud_Trtmt + (1|Soil_block/Soil_id/S.E_combo) + (1|exud_block/exud_id) +
(1|Plate/Row), data=h2, na.action=na.omit)
> summary(lm3)
Linear mixed model fit by REML. t-tests use Satterthwaite's method ['lmerModLmerTest']
Formula: log(CO2_C6) ~ Soil_Species * Soil_Trtmt * Exud_Species * Exud_Trtmt +      (1 | Soil_block/Soil_id/S.E_combo) + (1 |
exud_block/exud_id) +      (1 | Plate/Row)
   Data: h2

REML criterion at convergence: -6623.8

Scaled residuals:
     Min       1Q   Median       3Q      Max
-12.2766  -0.3883  -0.0061   0.4322   7.4982

Random effects:
 Groups                         Name        Variance  Std.Dev.
 S.E_combo:(Soil_id:Soil_block) (Intercept) 0.0076306 0.08735
 Row:Plate                      (Intercept) 0.0015452 0.03931
 Plate                          (Intercept) 0.0215173 0.14669
 exud_id:exud_block             (Intercept) 0.1810059 0.42545
 Soil_id:Soil_block             (Intercept) 0.0053319 0.07302
 exud_block                     (Intercept) 0.0005952 0.02440
 Soil_block                     (Intercept) 0.0067734 0.08230
 Residual                                   0.0041620 0.06451
Number of obs: 3200, groups:  S.E_combo:(Soil_id:Soil_block), 400; Row:Plate, 280; Plate, 35; exud_id:exud_block, 20; Soil_id:Soil_block, 20;
exud_block, 5; Soil_block, 5

Fixed effects:
                                                                         Estimate Std. Error        df t value Pr(>|t|)
(Intercept)                                                             1.270e-02  1.993e-01  1.839e+01   0.064  0.94986
Soil_SpeciesRumex                                                       1.111e-01  5.335e-02  1.821e+01   2.083  0.05161 .
Soil_TrtmtDrought                                                       6.322e-02  5.325e-02  1.807e+01   1.187  0.25050
Exud_SpeciesRumex                                                       5.424e-01  2.704e-01  1.558e+01   2.006  0.06259 .
Exud_TrtmtDrought                                                       9.359e-01  2.704e-01  1.558e+01   3.461  0.00333 **
Soil_SpeciesRumex:Soil_TrtmtDrought                                    -1.532e-01  7.540e-02  1.815e+01  -2.032  0.05702 .
Soil_SpeciesRumex:Exud_SpeciesRumex                                     8.444e-05  3.860e-02  3.254e+02   0.002  0.99826
Soil_TrtmtDrought:Exud_SpeciesRumex                                    -3.911e-02  3.795e-02  3.243e+02  -1.031  0.30344
Soil_SpeciesRumex:Exud_TrtmtDrought                                     2.098e-03  3.779e-02  3.243e+02   0.056  0.95576
Soil_TrtmtDrought:Exud_TrtmtDrought                                    -6.891e-03  3.769e-02  3.240e+02  -0.183  0.85506
Exud_SpeciesRumex:Exud_TrtmtDrought                                    -2.083e-01  3.825e-01  1.558e+01  -0.545  0.59379
Soil_SpeciesRumex:Soil_TrtmtDrought:Exud_SpeciesRumex                   1.108e-01  5.388e-02  3.246e+02   2.056  0.04055 *
Soil_SpeciesRumex:Soil_TrtmtDrought:Exud_TrtmtDrought                   4.364e-02  5.380e-02  3.246e+02   0.811  0.41779
Soil_SpeciesRumex:Exud_SpeciesRumex:Exud_TrtmtDrought                  -1.698e-02  5.408e-02  3.249e+02  -0.314  0.75370
Soil_TrtmtDrought:Exud_SpeciesRumex:Exud_TrtmtDrought                   2.408e-02  5.331e-02  3.240e+02   0.452  0.65179
Soil_SpeciesRumex:Soil_TrtmtDrought:Exud_SpeciesRumex:Exud_TrtmtDrought -8.747e-02  7.591e-02  3.245e+02  -1.152  0.25003
---
Signif. codes:  0 '***' 0.001 '**' 0.01 '*' 0.05 '.' 0.1 ' ' 1

Correlation matrix not shown by default, as p = 16 > 12.
Use print(x, correlation=TRUE)  or
    vcov(x)        if you need it

> plot(lm3)
> anova(lm3)
Type III Analysis of Variance Table with Satterthwaite's method
                                            Sum Sq  Mean Sq NumDF  DenDF F value    Pr(>F)
Soil_Species                              0.012549 0.012549     1  12.04  3.0152 0.1079678
Soil_Trtmt                                0.000026 0.000026     1  12.00  0.0063 0.9381518
Exud_Species                              0.021932 0.021932     1  15.34  5.2696 0.0361841 *
Exud_Trtmt                                0.079224 0.079224     1  15.34 19.0352 0.0005295 ***
Soil_Species:Soil_Trtmt                   0.008634 0.008634     1  12.00  2.0744 0.1753651
Soil_Species:Exud_Species                 0.007231 0.007231     1 324.71  1.7374 0.1884034
Soil_Trtmt:Exud_Species                   0.000502 0.000502     1 323.46  0.1206 0.7285615
Soil_Species:Exud_Trtmt                   0.000504 0.000504     1 323.25  0.1210 0.7281303
Soil_Trtmt:Exud_Trtmt                     0.000306 0.000306     1 324.10  0.0736 0.7863391
Exud_Species:Exud_Trtmt                   0.001472 0.001472     1  15.34  0.3537 0.5607083
Soil_Species:Soil_Trtmt:Exud_Species      0.013136 0.013136     1 324.17  3.1561 0.0765804 .
Soil_Species:Soil_Trtmt:Exud_Trtmt        0.000000 0.000000     1 323.84  0.0000 0.9980404
Soil_Species:Exud_Species:Exud_Trtmt      0.010214 0.010214     1 325.67  2.4541 0.1181860
Soil_Trtmt:Exud_Species:Exud_Trtmt        0.001115 0.001115     1 324.47  0.2679 0.6051159
Soil_Species:Soil_Trtmt:Exud_Species:Exud_Trtmt 0.005527 0.005527     1 324.50  1.3279 0.2500315
---
Signif. codes:  0 '***' 0.001 '**' 0.01 '*' 0.05 '.' 0.1 ' ' 1
```
